# Open-Windowed Hollow Carbon Architectures Enabling Low-Tortuosity Ion Transport for Supercapacitors

**DOI:** 10.3390/nano16100593

**Published:** 2026-05-12

**Authors:** Cunjing Wang, Xinzhong Yuan, Zhihua Ma, Huijun Liang, Pengfa Li

**Affiliations:** 1 School of Chemistry and Materials Engineering, Xinxiang University, Xinxiang, Xinxiang 453003, China; 2 School of Medicine, Xinxiang University, Xinxiang, Xinxiang 453003, China; 3 Graduate School, Xinxiang University, Xinxiang, Xinxiang 453003, China

**Keywords:** supercapacitors, hollow carbon architectures, tortuosity, molten-salt synthesis, hierarchical porous carbon

## Abstract

Carbon-based supercapacitors are fundamentally limited by the tortuosity of conventional microporous architectures, which restricts ion transport kinetics and impedes the full utilization of active sites, particularly under high-rate conditions. Herein, we report a molten-salt-assisted topological transformation strategy to fabricate nitrogen-doped hierarchical porous carbon (A-ZC) featuring a distinctive open-windowed hollow architecture. This design effectively mitigates the tortuosity of conventional microporous networks, creating low-resistance pathways that facilitate rapid ion flux and deep electrolyte penetration. Consequently, the symmetric supercapacitor delivers a high energy density of 11 Wh kg^−1^ at a power density of 250 W kg^−1^. Moreover, it exhibits outstanding cycling stability, retaining 98.9% of its initial capacitance after 20,000 cycles. By elucidating the correlation between salt-induced microstructural evolution and electrochemical kinetics, this work offers a robust blueprint for overcoming the intrinsic limitations of traditional porous architectures in high-performance energy storage.

## 1. Introduction

Electrochemical energy storage devices serve as pivotal components in the development of electric vehicles and smart grids, where performance metrics directly govern the operational efficiency of modern energy systems. Among various technologies, supercapacitors, utilizing an electric double-layer mechanism via physical adsorption/desorption, exhibit superior power density and life cycle compared to batteries; however, their practical deployment remains constrained by intrinsically limited energy density [1,2,3,4,5,6]. Although strategies such as heteroatom doping, pseudocapacitance introduction, and voltage window expansion have partially elevated the energy density limits, further breakthroughs heavily rely on carbon-based materials due to their tunable multidimensional structures, exceptional conductivity, and chemical stability [7,8,9,10,11,12,13,14]. Nevertheless, conventional porous carbon architectures face a critical challenge: while abundant micropores provide a high specific surface area (SSA) for charge storage, the excessive tortuosity within these confined channels severely impedes solvated ion transport. This hinders deep electrolyte infiltration, leaving numerous internal active sites inaccessible and ultimately restricting both energy density and rate capability [15].

To address the limitations of conventional microporous carbons, recent research has focused on breaking kinetic bottlenecks through precise structural engineering [16,17,18,19,20]. On the one hand, heteroatom doping enhances electrode wettability and introduces pseudocapacitance [21,22,23,24,25,26,27,28,29]. On the other hand, focusing on ion transport, the construction of hierarchical architectures featuring open-windowed hollow structures has emerged as a pivotal strategy to directly mitigate tortuosity and optimize ion transport pathways [28]. Such designs provide efficient channels for electrolyte penetration, preserving high surface area while utilizing mesoporous windows to shorten diffusion paths, thereby ensuring that a greater number of active sites participate in interfacial redox reactions. Regarding material synthesis, Metal–Organic Frameworks (MOFs), particularly ZIF-8, are ideal precursors for fabricating heteroatom-doped porous carbons due to their highly ordered crystalline structure and nitrogen-rich ligands [28,29]. To further refine pore connectivity and reduce production costs, molten salt-assisted method has proven to be a promising approach [30,31,32,33,34,35,36,37,38,39]. The salt serves a dual role as a high-temperature medium and an in situ oxygen barrier, preventing oxidation and thus enabling the cost-effective synthesis of high-quality carbon materials in ambient air [35,36,37,38,39].

In this work, we report a salt-sealed pyrolysis strategy to transform ZIF-8 into nitrogen-doped hierarchical porous carbon featuring a distinctive open-windowed hollow architecture. Specifically, the molten salt functions as a multifunctional medium: it not only protects the framework from oxidative degradation and suppresses excessive char formation during carbonization, but also directs structural evolution by exerting a templating effect that facilitates pore formation and stabilizes the hollow architecture. This study aims to precisely engineer the opening structure and pore connectivity via molten salt-assisted topological transformation. By elucidating a clear correlation between the microstructure evolution and electrochemical performance, this work provides new insights into designing carbon-based supercapacitors that effectively mitigate the tortuosity of the micropores to enable rapid ion transport.

## 2. Material and Methods

### 2.1. Material Preparation

All chemicals and reagents were purchased from Sinopharm Chemical Reagent Co., Ltd. (Shanghai, China). A solution of zinc nitrate hexahydrate (2.96 g) in methanol (100 mL) was prepared and stirred for 60 min. Separately, a solution of 2-methylimidazole (3.08 g) in methanol (100 mL) was prepared under identical conditions. The two solutions were then combined and stirred for an additional 30 min. The resulting mixture was allowed to stand undisturbed in air at room temperature for 24 h, during which time a white precipitate formed at the bottom of the beaker. The product was collected by centrifugation, washed several times with methanol, and dried in a forced-air oven (Yuhua Instrument, Gongyi, China) at 80 °C to obtain the ZIF-8 precursor.

The salt template consisting of KCl and NaCl (mass ratio 1:1) was first ground in an agate mortar. Subsequently, 0.15 g of ZIF-8 was thoroughly mixed with 4.5 g of the salt mixture in a 30 mL crucible. The crucible was covered and then heated in a muffle furnace (Yuhua Instrument, Gongyi, China) at 800 °C for 3 h to anneal the mixture. After cooling to room temperature, the product was washed repeatedly with 3 M HCl solution followed by deionized water to remove the salt template. The final sample, designated as A-ZC, was collected after drying in a forced-air oven.

For comparison, the control sample, denoted as N-ZC, was prepared by direct pyrolysis/carbonization of ZIF-8 at 800 °C for 3 h under a nitrogen flow (50 mL·min^−1^) in a tube furnace. The yield of sample A-ZC (prepared via the salt-sealed method in air) was 33%, whereas the yield of the control sample N-ZC (prepared under N_2_) was 50%.

### 2.2. Material Characterizations

The thermal behavior of ZIF-8, the salt mixture, and their composite was investigated using simultaneous thermogravimetric and differential scanning calorimetry (TG-DSC, NETZSCH, Selb, Germany) in an air atmosphere with a heating rate of 5 °C min^−1^ from room temperature to 900 °C. The morphology and microstructure of the samples were characterized by scanning electron microscopy (SEM, FEI Quanta FEG 250, Thermo Fisher Scientific, Hillsboro, OR, USA) and transmission electron microscopy (TEM, JEM-2100, JEOL Ltd., Tokyo, Japan) equipped with elemental mapping. Structural analysis was carried out using X-ray diffraction (XRD, Bruker D8 Advance, Bruker Corp., Billerica, MA, USA) and Raman spectroscopy (LabRAM HR, HORIBA Scientific, Kyoto, Japan). Surface chemistry and elemental composition were probed by X-ray photoelectron spectroscopy (XPS, PHI 5700 ESCA System, Physical Electronics, Chanhassen, MN, USA). Specific surface area (SSA) and pore structure were characterized by N_2_ adsorption–desorption isotherms at 77 K using a Quantachrome NOVA 2000e analyzer NOVA 2000e analyzer (Quantachrome Instruments, Boynton Beach, FL, USA). Prior to measurement, all samples were degassed under vacuum at 200 °C for 12 h to remove any adsorbed moisture and impurities. The Brunauer–Emmett–Teller (BET) method was employed to calculate the specific surface area within a relative pressure (*P*/*P*_0_) range of 0.05–0.30. The total pore volume (*V*_total_) was estimated from the amount of N_2_ adsorbed at a relative pressure of *P*/*P*_0_ ≈ 0.99. Pore size distribution (PSD) was derived from the adsorption branch of the isotherm using the Non-local Density Functional Theory (NDFT) model for carbon slit pores.

### 2.3. Electrochemical Measurements

Working electrodes were fabricated by uniformly coating a slurry of porous carbon, carbon black, and polytetrafluoroethylene (PTFE) binder (80:15:5 by weight) onto stainless steel mesh current collectors, yielding an active mass loading of approximately 3 mg cm^−2^. Electrochemical measurements, including cyclic voltammetry (CV) and electrochemical impedance spectroscopy (EIS), were performed on a CHI660C workstation (Chenhua, Shanghai, China) using a standard three-electrode setup in 1 mol L^−1^ H_2_SO_4_ electrolyte. In the three-electrode system, the working, counter, and reference electrodes were the carbon electrode, a platinum foil, and an Ag/AgCl electrode, respectively. CV measurements were carried out at scan rates from 10 to 100 mV s^−1^ over a potential window of 0–1 V vs. Ag/AgCl. EIS was recorded over the frequency range of 100 kHz to 0.1 Hz with an AC amplitude of 5 mV. Furthermore, symmetric supercapacitors were assembled using a glassy fibrous separator and evaluated in the same electrolyte using a LAND CT2001A battery test system (LAND Electronic Co., Ltd., Wuhan, China). The specific capacitance (*C*_g_, F g^−1^) of single electrode in two electrode was calculated according to equation

(1) $$Cg=4\times \frac{I\Delta t}{m\Delta V}$$

where *I* (A) is the constant charge/discharge current, Δ*t* (s) is discharge time, Δ*V* is the voltage window during the discharge process and *m* (g) is the total mass of active materials in the two electrodes. The gravimetric energy density *E*_g_ (Wh kg^−1^), normalized to the total mass of the active materials in both electrodes, was estimated using the following equation

(2) $$E_{g}=\frac{Cg\Delta V}{8\times 3.6}$$

The gravimetric power density *P*_g_ (W kg^−1^), based on the total discharge time, was calculated according to the following equation

(3) $$P_{g}=\frac{3600E_{g}}{\Delta t}$$

## 3. Results and Discussion

**Figure 1:**
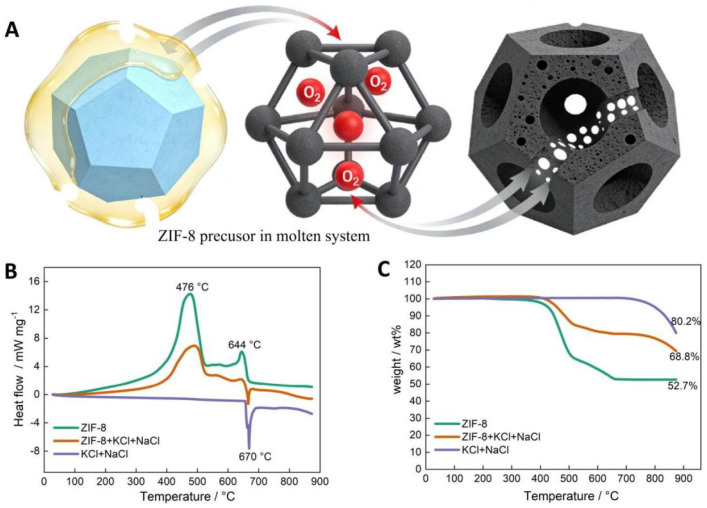
Structural and thermal evolution of ZIF-8 during molten salt processing. (**A**) Schematic of the molten salt-mediated formation of a porous hollow architecture, involving spatial confinement and oxidative etching. (**B**) DSC profiles and (**C**) TG curves for ZIF-8, ZIF-8 + KCl–NaCl mixture, and pure KCl–NaCl.

The structural evolution of ZIF-8 during molten salt processing was elucidated by combining schematic modeling with thermal analysis (DSC-TG), as summarized in Figure 1. This approach reveals a three-stage transformation pathway and the associated thermal behavior. Figure 1A schematically depicts the three-stage transformation: (i) The crystalline ZIF-8 precursor is spatially confined within a molten salt matrix (yellow region) during carbonization, yielding a stabilized intermediate; (ii) at the atomic scale, the molten salt mediates the oxidative etching of the nascent carbon framework by O_2_ (red spheres), which is pivotal for generating intraparticle voids; (iii) the final product evolves into a porous, hollow architecture with interconnected voids.

Complementary thermal analysis (Figure 1B, DSC; Figure 1C, TG) reveals distinct energetic and mass loss behaviors across systems. In the DSC profile (Figure 1B), the ZIF-8 + KCl + NaCl system exhibits a sharp exothermic peak at 476 °C, consistent with framework crystallization or rearrangement, followed by a second peak at 644 °C corresponding to further structural evolution. In contrast, the pure ZIF-8 and KCl + NaCl systems display distinct thermal responses, highlighting the role of the molten salt in modulating ZIF-8’s thermal behavior. TG analysis (Figure 1C) further shows distinct mass loss profiles: while the ZIF-8 derived systems exhibit a characteristic two-step mass loss, the pure salt shows a single-step dehydration. The first step (≤300 °C) corresponds to adsorbed solvent/water desorption, and the second step (300–700 °C) is attributed to ZIF-8 framework decomposition. Notably, the composite retains 68.8% of its mass at 900 °C in air, outperforming the theoretical prediction of 66.45% for a simple 1:1 mixture. This positive deviation points to a non-additive, synergistic stabilization mechanism. Unlike inert diluents, the molten salt matrix functions as an oxygen diffusion barrier, creating a confined microenvironment around the ZIF-8 framework. We propose that this encapsulation moderates the oxidative degradation of the organic ligands, effectively preserving the carbonaceous matrix. This confinement ensures the structural integrity of the resulting carbon scaffold, thereby enhancing the overall thermal robustness of the composite system.

**Figure 2:**
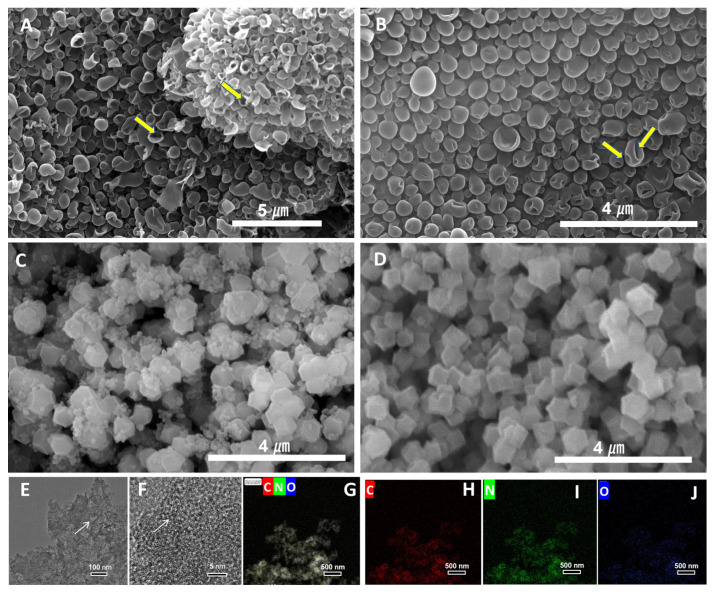
Morphological and microstructural characterization. SEM images of A-ZC (**A**,**B**), N-ZC (**C**), ZIF-8 (**D**). TEM (**E**), HRTEM (**F**). Elemental mapping (**G**–**J**) of C, N, and O in A-ZC; red, green, and blue represent C, N, and O, respectively. Yellow arrows in SEM (**A**,**B**) indicate broken particle fragments; white arrows in TEM (**E**,**F**) indicate the interconnected carbon ligaments forming the internal pore network.

SEM images (Figure 2A,B) reveal that the carbonized A-ZC sample consists of a uniform population of submicron spherical architectures (~300–500 nm). Crucially, broken particle fragments (yellow arrows) confirm hollow interiors encapsulated by thin carbon shells, which are characterized by open windows and surface pores. This unique architecture is a direct result of the molten salt-mediated oxidative etching process (Figure 1A), where the salt matrix acts as a physical template while trace oxygen etches the carbon framework. In contrast, N-ZC synthesized under inert conditions forms dense, solid-like aggregates (Figure 2C), underscoring that the molten salt matrix serves as a physical template while trace oxygen simultaneously etches the carbon framework, converting the original ZIF-8 polyhedra (Figure 2D) into hollow architectures with open windows.

Turning to the nanoscale structure, Figure 2E reveals a hollow, cage-like architecture with thin, porous walls, consistent with the removal of the Zn-based core. Figure 2F provides a higher-magnification view, showing the internal network of mesopores and micropores formed by the interconnected carbon ligaments (white arrows). Notably, no crystalline Zn particles or metallic domains are observed. HAADF-STEM and elemental mapping (Figure 2G–J) further confirm the complete removal of Zn via thermal evaporation and HCl washing, validating its role as a sacrificial template for pore generation. Uniform distributions of C, N, and O throughout the metal-free carbon framework ensure high structural continuity and electrical conductivity. Collectively, these results establish that the molten salt-mediated oxidative etching strategy effectively transforms ZIF-8 into an open-windowed, hollow carbon architecture. This unique structure facilitate rapid mass transport and provide accessible active sites, offering distinct advantages for electrochemical energy storage applications.

**Figure 3:**
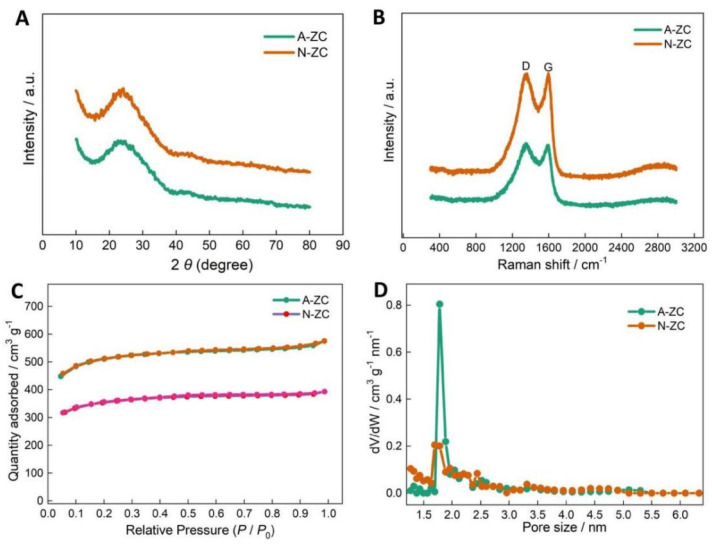
Structural and textural characterization of A-ZC and N-ZC. (**A**) Powder XRD patterns, (**B**) Raman spectra; D and G bands denote disordered and graphitic carbon, respectively. (**C**) N_2_ adsorption–desorption isotherms at 77 K; the orange line represents adsorption, and the blue line represents desorption of A-ZC, and the purple line represents the adsorption and the pink line represents the desorption of N-ZC. (**D**) DFT pore size distributions.

Figure 3A–D collectively elucidate the structural and textural evolution of the samples. The XRD patterns (Figure 3A) of both samples show a broad (002) diffraction peak at ~23°, typical of disordered carbonaceous materials. Notably, the absence of a well-defined (110) peak at ~43° in either sample indicates limited long-range order, consistent with the HRTEM observations. Raman spectra (Figure 3B) and quantitative analysis (Table 1) reveal a slightly elevated *I*_D_/*I*_G_ ratio for A-ZC (1.04) compared to N-ZC (1.02), confirming a marginally higher defect density. These structural characteristics suggest abundant edge-site exposure and lattice distortions in the carbon framework, which typically facilitate ion and electron accessibility. N_2_ adsorption–desorption isotherms (Figure 3C) and pore size distributions (Figure 3D) quantitatively corroborate the differences in surface area and porosity. As summarized in Table 1, A-ZC exhibits a moderate yet distinct increase in BET surface area (1589 m^2^ g^−1^) and total pore volume (0.89 cm^3^ g^−1^) compared to N-ZC (1103 m^2^ g^−1^ and 0.72 cm^3^ g^−1^, respectively). Moreover, the corresponding pore size distributions (Figure 3D) reveal distinct structural signatures. While both samples exhibit pores within the 1–6 nm range, A-ZC is dominated by a sharp, intense peak centered at 1.8 nm, indicative of a highly uniform pore structure. In contrast, N-ZC shows a weak and broad distribution across the same range, reflecting a disordered pore structure with low size uniformity. This difference arises from the unique structural evolution of A-ZC in molten salt, which integrates intrinsic uniform micropores with hollow interiors and open windows, thereby creating a hierarchical pore architecture that facilitates efficient ion accessibility and diffusion.

**Figure 4:**
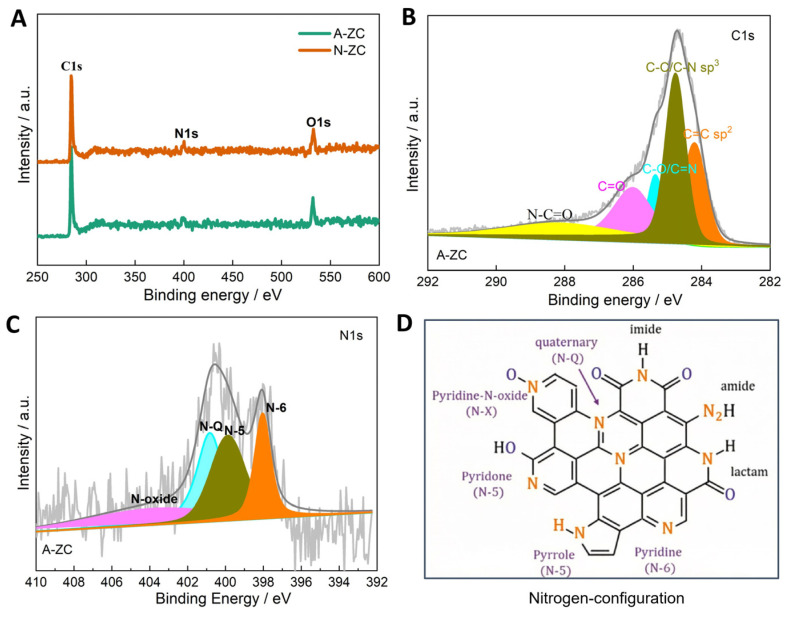
XPS analysis. (**A**) Survey spectra of A-ZC and N-ZC, (**B**,**C**) High-resolution C 1s and N 1s spectra of A-ZC; N-6, N-5, N-Q, and N-oxide correspond to pyridinic, pyrrolic, graphitic, and oxidized nitrogen species, respectively. (**D**) schematic of nitrogen configurations in the carbon matrix.

The chemical composition of A-ZC and I-ZC was analyzed by XPS. The survey spectra (Figure 4A) confirm the presence of C, N, and O in both samples, with characteristic peaks at 284.4, 399.9, and 532.0 eV, respectively. Quantitatively, A-ZC exhibits a composition of C 79.71 at.%, N 8.95 at.%, and O 11.34 at.%, whereas N-ZC consists of C 83.27 at.%, N 9.61 at.%, and O 7.11 at.% (Table 1). High-resolution C1s spectra of A-ZC (Figure 4B) are deconvoluted into five components at 284.3, 285.1, 286.2, 287.5, and 288.3 eV, corresponding to C=C, C–C/C–N, C–N/C–O, C=O, and N–C=O groups [40]. Similarly, the N 1s spectrum (Figure 4C) is resolved into four distinct chemical states: pyridinic-N (N-6, 398.2 eV), pyrrolic-N (N-5, 399.8 eV), graphitic-N (N-Q, 401.1 eV), and N-oxide (403.8 eV) [41], with their structural configurations illustrated in Figure 4D [16]. This chemical heterogeneity, characterized by the coexistence of multiple nitrogen configurations rather than a single dominant species, is expected to facilitate enhanced electrochemical kinetics and structural stability [40,41,42,43,44].

**Figure 5:**
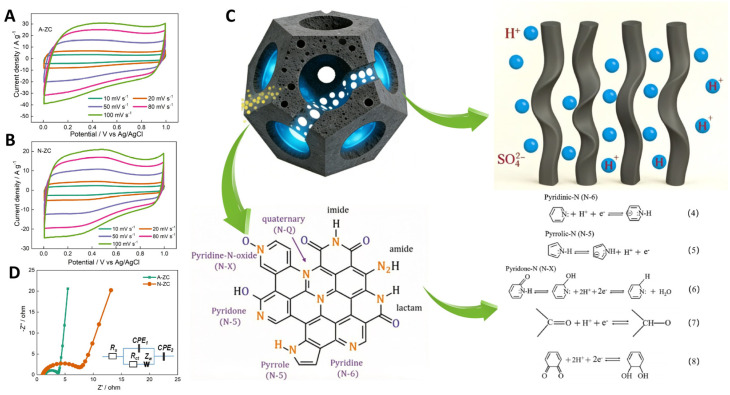
Electrochemical performance and structural mechanism. (**A**,**B**) CV curves of A-ZC and N-ZC at 10–100 mV·s^−1^. (**C**) Schematic illustration of the open-windowed hollow architecture enabling efficient ion transport and full utilization of pseudocapacitive active sites. (**D**) Nyquist plots with embedded equivalent circuit models.

To evaluate the practical implications of these structural advantages, we systematically compared the electrochemical performance of A-ZC and N-ZC. Figure 5A,B display the CV curves of A-ZC and N-ZC electrodes at scan rates from 10 to 100 mV·s^−1^ in a three electrode system. Both electrodes exhibit quasi-rectangular CV profiles characteristic of electric double-layer capacitive behavior, accompanied by symmetric redox humps between 0.2 and 0.6 V. As clearly seen in Figure 5A,B, A-ZC delivers significantly higher current densities than N-ZC. As shown in Figure 5C, this superiority of A-ZC is attributed to the open-windowed hollow architecture, which shortens ion diffusion paths and ensures high accessibility to both the internal surface and the pore interior, thereby facilitating rapid electrolyte infiltration and maximizing the utilization of the total specific surface area. Additionally, the same structural advantage enables full activation of heteroatom functionalities via reversible redox reactions of nitrogen and oxygen species [15]. Specifically, the nitrogen configurations contribute distinct pseudocapacitive mechanisms in the acidic electrolyte: pyridinic-N (N-6) engages in proton-coupled electron uptake (Equation (4)), pyrrolic-N (N-5) contributes via reversible oxidation (Equation (5)), and pyridone-N (N-X) undergoes proton-coupled electron transfer (Equation (6)). Similarly, carbonyl and quinone groups participate in proton-coupled electron transfer, producing supplementary faradaic humps (Equations (7) and (8)) [15]. Consequently, the open architecture of A-ZC not only provides abundant active sites but also ensures efficient utilization of these nitrogen and oxygen sites, leading to its superior specific capacitance.
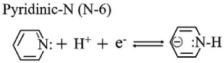
(4)
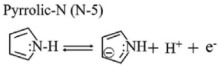
(5)
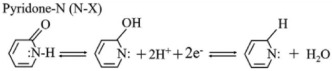
(6)
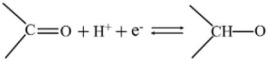
(7)
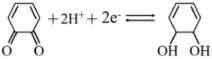
(8)

Figure 5D presents the Nyquist plots for both samples, with the embedded equivalent circuit diagram used for data fitting. In this circuit, *R*_s_ represents the solution resistance; *R*_ct_ corresponds to the charge transfer resistance at the electrode/electrolyte interface; CPE (constant phase element) describes the deviation from ideal capacitive behavior due to surface roughness and porosity; and *Z*_W_ denotes the Warburg impedance related to ion diffusion [36,37,38,39]. As quantitatively revealed by the fitting results (Table 2), A-ZC exhibits a slightly higher solution resistance (*R*_s_, 1.57 Ω) compared to N-ZC (1.13 Ω). This marginal increase is likely attributed to the larger specific surface area and more developed porous structure of A-ZC, which can lead to incomplete electrolyte infiltration at the initial stage of the EIS measurement, thereby affecting the high-frequency intercept. Notably, the high-frequency semicircle of A-ZC is significantly smaller, yielding a reduced charge transfer resistance (*R*_ct_) of 2.25 Ω compared to 6.86 Ω for N-ZC. Furthermore, the shorter Warburg region in the mid-frequency range indicates a lower Warburg impedance (*Z*_W_) of 0.38 Ω for A-ZC relative to 0.49 Ω for N-ZC. This confirms more facile ion diffusion through its interconnected porous network. Moreover, the fitting results reveal distinct interfacial properties. While the CPE magnitudes (CPE_1_) are comparable and reflect the overall double-layer capacitance, the significantly higher CPE exponent (CPE_2_, n) for A-ZC (0.94) compared to N-ZC (0.87) suggests that the surface of A-ZC exhibits more ideal double-layer capacitive behavior, indicating a more homogeneous and less rough electrode/electrolyte interface, which is consistent with the reduced charge transfer resistance observed for A-ZC.

**Figure 6:**
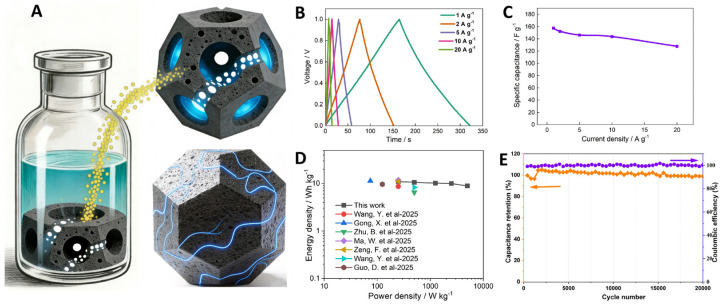
Structural mechanism and electrochemical performance. (**A**) Schematic showing the mitigation of tortuosity by the open-windowed hollow architecture. (**B**) GCD curves, (**C**) rate capability, (**D**) Ragone plot [44,45,46,47,48,49,50], and (**E**) cycling stability at 5 A·g^−1^ of A-ZC//A-ZC symmetric device.

Leveraging the structural design outlined in Figure 6A, we constructed an A-ZC//A-ZC symmetric device. Figure 6B displays nearly ideal, isosceles-triangular GCD profiles across a wide current density range (1–20 A·g^−1^), indicative of typical EDLC-type behavior and good charge/discharge reversibility. Correspondingly, the device achieves a high specific capacitance of 158 F·g^−1^ at 1 A·g^−1^ and retains 128 F·g^−1^ even at 20 A·g^−1^ (Figure 6C), based on the total mass of the active materials in both electrodes, underscoring its exceptional rate capability. Such outstanding performance stems from a synergistic combination of factors: (1) the high specific surface area (SSA) provides a large reservoir of charge accommodation sites; (2) the unique hollow architecture with open windows facilitates rapid electrolyte infiltration and shortens ion diffusion paths, ensuring full utilization of the internal surface area; and (3) the synergy of the interconnected pore network ensures efficient accessibility of redox-active sites, thereby enhancing pseudocapacitance. Building on these structural and kinetic advantages, as shown in the Ragone plot (Figure 6D), the device achieves an energy density of 11 Wh·kg^−1^ at 250 W·kg^−1^, based on the total mass of the active materials in both electrodes, significantly outperforming commercial activated carbon (typically 5–8 Wh·kg^−1^) and either surpassing or matching the leading reported values for symmetric carbon-based supercapacitors in aqueous electrolytes [44,45,46,47,48,49,50]. Furthermore, the device exhibits outstanding long-term cyclability. After 20,000 cycles at 5 A·g^−1^, it retains 98.9% of its initial capacitance with a stable Coulombic efficiency of nearly 100% (Figure 6E), underscoring the structural and electrochemical stability of the open-hollow design under prolonged operation.

## 4. Conclusions

In summary, this work develops a molten-salt-assisted topological transformation strategy to synthesize nitrogen-doped hierarchical porous carbon (A-ZC) featuring a distinctive open-windowed hollow architecture. It is demonstrated that this design effectively mitigates the tortuosity of micropores, creating low-resistance pathways that facilitate rapid ion transport and deep electrolyte penetration. This structural advantage, synergistically coupled with redox-active nitrogen and oxygen functionalities, maximizes the utilization of both the accessible surface area for electric double-layer capacitance and the exposed heteroatom-active sites for Faradaic pseudocapacitance. Consequently, the A-ZC//A-ZC symmetric device delivers a high energy density of 11 Wh kg^−1^ at a power density of 250 W kg^−1^ on the active-mass basis, and exhibits remarkable long-term cycling stability with 98.9% capacitance retention over 20,000 cycles. While the current energy density is still limited by the narrow voltage window of aqueous electrolytes, this study establishes a robust platform for future optimization. By expanding the operating voltage via neutral/organic electrolytes or constructing asymmetric configurations with battery-type cathodes (e.g., MnO_2_), the energy density of the A-ZC-based system is expected to be significantly boosted. Ultimately, this work provides a clear structure–property blueprint for designing high-performance carbon-based electrodes, thereby expanding the design space for next-generation energy storage devices.

## Figures and Tables

**Table 1:** Summary of textural properties, Raman spectroscopy, and XPS elemental analysis for A-ZC and N-ZC.

Sample	*S*_BET_ (m^2^ g^−1^)	*V*_total_ (cm^3^ g^−1^)	PSD (nm)	*I*_D_/I_G_	C (at.%)	N (at.%)	O (at.%)
A-ZC	1589	0.89	Sharp (~1.8)	1.04	79.71	8.95	11.34
N-ZC	1103	0.72	Broad (~1.8)	1.02	83.27	9.61	7.11

**Table 2:** EIS fitting parameters for A-ZC and N-ZC (values ± error); errors represent 95% confidence intervals from nonlinear least-squares fitting.

Parameter	A-ZC	N-ZC	Description
*R*_s_ (Ω)	1.57 ± 0.05	1.13 ± 0.07	solution resistance
*R*_ct_ (Ω)	2.25 ± 0.12	6.86 ± 0.30	charge transfer resistance
CPE_1_ (F s n^−1^)	0.042 ± 0.003	0.037 ± 0.003	CPE magnitude
CPE_2_ (n)	0.94 ± 0.02	0.87 ± 0.03	CPE exponent
*W* (S s^−0.5^)	0.38 ± 0.03	0.49 ± 0.04	Warburg impedance

## Data Availability

The original contributions presented in this study are included in the article. Further inquiries can be directed to the corresponding authors.
